# Therapeutic Sensations: A New Unifying Concept

**DOI:** 10.1155/2020/7630190

**Published:** 2020-08-06

**Authors:** Florian Beissner

**Affiliations:** Somatosensory and Autonomic Therapy Research, Institute for Neuroradiology, Hannover Medical School, Hannover, Germany

## Abstract

Physical sensations of tingling, warmth, dull pain, and heaviness are a common phenomenon in mind-body interventions, such as acupuncture, hypnotherapy, osteopathy, qigong, meditation, and progressive muscle relaxation. Even though there are striking parallels between sensations produced by many different interventions, no attempt has yet been made to understand them from a unifying perspective that combines information from different therapies and practices. Therefore, this narrative systematic review introduces the concept of therapeutic sensations and summarizes studies of their sensory quality, bodily topography, and the meaning that patients attach to them. Furthermore, it highlights the essential role of therapeutic sensations in the development of vital energy concepts, such as *qi*, *prana*, *pneuma*, and *orgone,* in various traditional medicine systems, body-oriented psychotherapy, and so-called energy medicine. Finally, the assessment of therapeutic sensations may help to gain a deeper understanding of such concepts, finding a common language between scientists, patients and practitioners, and bridging the wide gap between materialistic and vitalistic views.

## 1. Introduction

“A sensation of warmth and tingling in the limbs. It's usually during the treatment... but not right off. It takes a few minutes...”

Patient after sham acupuncture treatment [1].

“I always felt the heat coming out of the therapist's hands, in every session I felt.”

Nursing student after therapeutic touch treatment [2].

“It's quite a bizarre sensation that I've never had before.”

Patient after acupuncture treatment [3].

“You can feel the wind going round in your body ... it was like she was chasing wind round my body.”

Patient after therapeutic touch treatment [4].

”I feel a sense of peace, calm, and warmth all over my body when I walk into the room. It's a tingling feeling that flows like a stream over me.”

Practitioner after Taijiquan session [5].

“I had the impression that I could direct a subtle tingling, a flow of energy, through my entire body.”

Practitioner of Vipassana meditation [6].

Complex bodily sensations like the ones described above are a common phenomenon in mind-body interventions, such as acupuncture, hypnotherapy, osteopathic medicine, qigong, meditation, and progressive muscle relaxation. Well-known examples include the feeling of heaviness and warmth in autogenic training [7], the needling sensation in acupuncture [8], enhanced touch sensations in ritual touch healing [1], and energetic flow sensations in body-oriented psychotherapy, qigong, or the internal martial arts [5]. An illustrative account comes from Wilhelm Reich, one on the forefathers or body-oriented psychotherapy [9]: “The loosening of the rigid muscular attitudes produced peculiar body sensations in the patients: involuntary trembling and twitching of the muscles, sensations of cold and hot, itching, the feeling of pins and needles, prickling sensations, the feeling of having the jitters, and somatic perceptions of anxiety, anger, and pleasure.”

At present, there is no generally accepted term for this kind of sensations. Therefore, the author proposes the term *therapeutic sensations* for them (see Definition 1). It is motivated by the observation that such sensations occur in a therapeutic or self-cultivation context and that the person experiencing them often associates them with concepts of vital energy, well-being, or healing.

**Definition 1 .**

Therapeutic sensations: complex physical sensations in the context of mind-body interventions and exercises that are experienced by the person exercising or being treated, by the practitioner, or by both.

Common therapies that elicit therapeutic sensations include acupuncture [8, 10–13], acupressure [14, 15], moxibustion [16], cupping [17], and other related techniques. However, they also occur in various other interventions, such as massage [18], cryotherapy [19], pulsed ultrasound [20], trigger point therapy [21], body-oriented psychotherapy [9, 22], transcutaneous electrical nerve stimulation [23], low-level laser stimulation [24, 25], auriculotherapy [26], hypnotherapy [27], osteopathic medicine [28], Reiki [29], therapeutic touch [5, 30], charismatic healing [31], meditation [6, 32, 33], and somatic bodywork methods [34].

Furthermore, a growing body of evidence shows that therapeutic sensations are elicited by placebo stimulation as reported for placebo laser stimulation [24, 25, 35], placebo TENS [36], and the topical application of placebos [37]. Finally, several studies have reported sham acupuncture to elicit therapeutic sensations [38, 39].

Similar sensations exist outside the realm of therapy or exercise. They include attention-related sensations [37, 40–42] that arise during self-focused bodily attention, chills or frisson [43, 44], a goose bumps type of skin sensation often triggered by emotional music, and the so-called autonomous sensory meridian response (ASMR), a tingling, static-like sensation across the scalp and the back of the neck [45, 46]. At the end of the 18th century, Immanuel Kant coined the term *vital sensations* (Vitalempfindungen) for such body experiences at the border between psyche and soma and described them very vividly: “The sensation of warmth and cold, including the sensation aroused by the mind (e.g., through quickly rising hope or fear), belong to the vital sense. The shudder that seizes man himself at the thought of the sublime, and the terror with which the nurse's tales drive children to bed late at night, belong to the latter; they penetrate the body to the extent that there is life in it.” ([47], translation by the author).

Despite the striking similarity of therapeutic sensations in different interventions and exercises, no attempt has been made so far to understand their characteristics from a unifying perspective that brings together information from various therapies and practices. Therefore, the aim of this review article is to provide a phenomenological overview of the existing evidence on therapeutic sensations. Methodologically, the article is a narrative systematic review (see Supplementary Methods, available here) combining the advantages of these two complementary review approaches [48]. Thematically, it covers three phenomenological domains: sensory qualities of therapeutic sensations, their bodily topography, and the meaning that patients attach to them. Furthermore, the author discusses the essential role of therapeutic sensations in the quest to scientifically assess concepts of vital energy in traditional medicine systems, body-oriented psychotherapy, and the so-called energy medicine.

## 2. Somatosensory Qualities of Therapeutic Sensations

The first phenomenological domain of therapeutic sensations includes somatosensory qualities, that is, verbal descriptions by the person experiencing them. These are either collected as free responses or by having the person choose from a list of predefined descriptors. Six examples obtained with the former method are shown in the beginning of this article. Naturally, they exhibit a high level of variability, and similar reports are often mixtures of sensory, evaluative, and affective descriptions (e.g., [1]). Studies using free responses commonly apply qualitative methods [49], such as grounded theory, and are indispensable to grasp the phenomenon of therapeutic sensations in its entirety. In contrast, lists of predefined descriptors allow for quantitative analyses, for example, by comparing frequencies of individual descriptors or using data mining approaches, such as factor analysis [8, 51, 52]. However, one must be careful to avoid bias, when selecting descriptors, a caveat best illustrated by the following example. In 1989, Vincent and colleagues developed the first questionnaire to assess acupuncture-related therapeutic sensations [8]. It consisted of twenty descriptors from the McGill Pain Questionnaire [52], selected by a group of acupuncturists. Later, Park and colleagues added five additional descriptors to the list [10]. The general approach, however, was criticized later by MacPherson and Asghar for starting from a pain questionnaire [53]. According to their argument, the selection of descriptors is likely to reflect only a limited facet of therapeutic sensations and may, thus, show suboptimal discrimination of pain and other sensory qualities. These authors then applied a Delphi method with acupuncture experts and derived seven sensations typically associated with acupuncture-related therapeutic sensations as well as nine describing pain. White and colleagues added the missing patients' descriptions in this approach [50]. They introduced a new scale based on previous literature, expert opinions and patients' experiences, which led to the Southampton Needle Sensation Questionnaire (SNSQ), a widely used seventeen-item score [50]. Using a similar approach, Kim and colleagues developed the Acupuncture Sensation Questionnaire (ASQ) that adds the new category of (general) bodily sensations to the already large group of sensations at the needle site [54]. These describe complex sensations, like “refreshing or relieving,” “activated blood circulation,” “activated digestion with intestinal movement,” and “surging opening flow of a stuffed or choked feeling.” Several other groups have developed similar questionnaires (see [55] for a review).

All questionnaires on somatosensory qualities of therapeutic sensations have in common that they report one or more of the following variables: intensity ratings of individual descriptors measured with some form of analogue scale (e.g., [12, 56]), frequencies of individual descriptors (e.g., [57, 58]), or some form of validity or appropriateness of individual descriptors based on expert ratings (e.g., [53, 54]). This review focuses on descriptor frequencies, thus excluding intensities and expert ratings.

### 2.1. Similar Qualities across Different Interventions

To compare the somatosensory qualities of therapeutic sensations across interventions, the author conducted a systematic literature search (see Supplementary Methods), which yielded the following 18 studies. Abundant literature exists for manual acupuncture [11, 20, 50, 58–65], while only isolated studies are available for other interventions and exercises. In addition to acupuncture, the following interventions were included in the comparison: transcutaneous electrical nerve stimulation (TENS) [23, 61], pulsed focused ultrasound [20], electroacupuncture [61, 66], therapeutic touch [57], low-level laser stimulation [3, 35], focused bodily attention [37, 42], and placebo stimulation with a deactivated laser or topical placebo solution [35, 37, 65].

**Figure 1:**
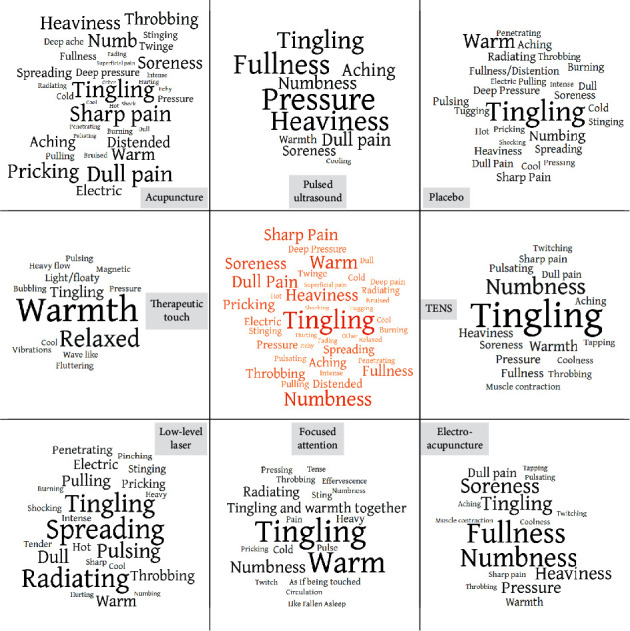
Somatosensory qualities of therapeutic sensations in various interventions (outer ring) and their average (centre). Each word cloud represents the descriptor profile of an intervention with relative descriptor frequency encoded by font size; that is, larger words are more commonly used than smaller ones. For each intervention, the centre of the cloud shows the most common descriptor. There is a broad similarity between the interventions regarding their therapeutic sensations as evidenced by the most common descriptors “tingling,” “warm,” “fullness,” “soreness,” “heaviness,” “numbness,” “pressure,” and “dull pain” appearing in most of the word clouds.

Therapeutic sensations from different interventions showed a broad similarity (see Figure 1) as evidenced by “tingling” and “warm” appearing in the descriptor profiles of all eight interventions, “heaviness,” “numbness,” “pressure,” “pulsating,” and “cool” in all but one and “throbbing” in all but two descriptor profiles. The most common descriptors with their frequencies in brackets were “tingling” (735), “numbness” (528), “warm” (479), “dull pain” (431), “heaviness” (417), “soreness” (399), “sharp pain” (389), “pricking” (321), “fullness” (317), “throbbing” (267), “aching” (249), “spreading” (214), and “pressure” (204).

### 2.2. Painful, Thermal, and Paresthetic Sensations

The multitude of descriptors encountered in therapeutic sensations makes neurophysiological characterization of the phenomenon a challenging task. The five most common descriptors alone cover somatosensory experiences of temperature (“warm”), innocuous sensations (“heaviness”), pain (“dull pain”), and paresthesia (“tingling”, “numbness”). The fact that many descriptors cannot be unambiguously assigned to a single somatosensory submodality further complicates the situation.

Valuable insights come from studies of acupuncture-related therapeutic sensations, where several groups have analyzed descriptor profiles and thereby added to our understanding of different sensation components. Using principal component analysis, Vincent and colleagues reported that one of the seven components detected by this approach comprised the descriptors “pulling,” “numb,” “heavy,” “dull,” and “aching” and likely corresponded to the acupuncture-related therapeutic sensations widely known as deqi (see below for detailed discussion) [8]. Other components found in their analysis described an overall pain intensity dimension and further pain experiences sometimes incorporated in descriptions of deqi. The results were largely replicated by Yin and colleagues, who, however, found a four-factor solution [51]. Building on the results of Vincent and colleagues but using their modified questionnaire, White and colleagues identified two components of acupuncture-related therapeutic sensations using factor analysis [50]. They called these components “aching deqi” and “tingling deqi.” The former described painful sensations felt during needling and comprised descriptors, such as “deep ache,” “dull ache,” “heavy,” “pressure,” and “stinging,” while the latter consisted of terms, such as “tingling,” “warm,” “numb,” “spreading,” and “throbbing,” thus, describing sensations generally not considered painful.

**Figure 2:**
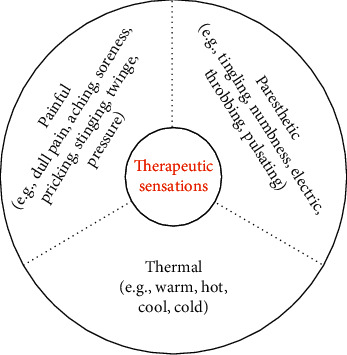
Therapeutic sensations can be broadly classified into three different categories based on the most common verbal descriptors. Since sensory physiology and perception psychology may differ between categories, such classification can inform future studies on the mechanism behind therapeutic sensations and their putative therapeutic role. There may be further categories (e.g., tactile and visceral) that have gone unnoticed due to the relative lack of descriptor-based questionnaires for these domains.

We can infer from these studies that therapeutic sensations come in a *painful* and *nonpainful* variety. However, since warm and tingling are very different sensations from a neurophysiological point of view, it may be more appropriate to distinguish three main categories: (1) *painful*, as described by “dull pain,” “soreness,” “sharp pain,” “aching,” and so on, (2) *paresthetic*, as described by words such as “tingling,” “numbness,” or “throbbing,” and (3) *thermal*, as described by “warm,” “hot,” “cold,” and so on (Figure 2).

## 3. The Topography of Therapeutic Sensations

Another salient feature of therapeutic sensations is that they are not restricted to the body region, where stimulation takes place. Instead, their topography includes sensations spreading from the site of stimulation or appearing in remote body regions as evidenced by the reports given in the introduction. Here, subjects report sensations “all over my body,” “going round in your body,” or flowing “like a stream over me” indicating widespread and sometimes propagating sensations with intricate patterns. Furthermore, the high prevalence of the attributes “spreading” and “radiating” in verbal descriptions of therapeutic sensations indicates that such experiences are the rule rather than the exception.

**Figure 3:**
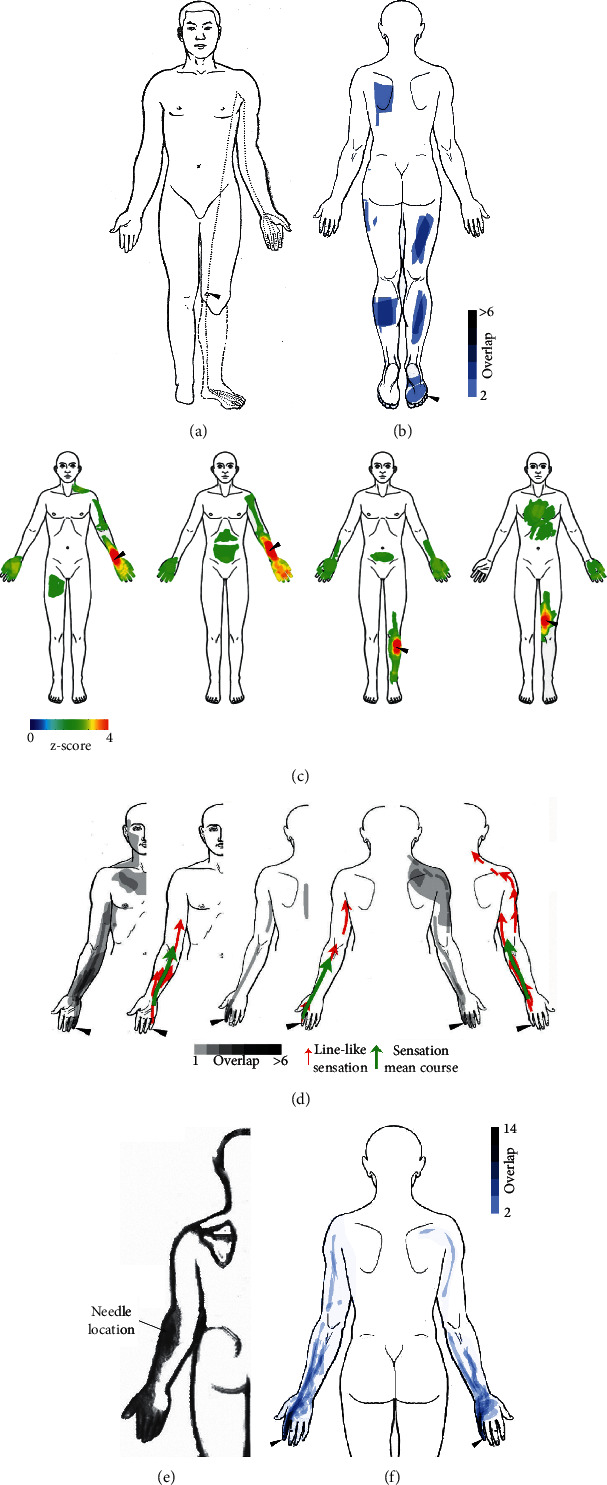
The topography of therapeutic sensations in various interventions. In each bodily map, the arrowhead marks the region of stimulation. (a) Electroacupuncture with sensations extending to the arm and the phantom limb in an acquired amputee, (b) placebo stimulation with a mock laser, (c) manual acupuncture of four different acupuncture points, (d) low-level laser stimulation under sensory deprivation, where linear sensations (red arrows) have been averaged (green arrows), (e) manual acupuncture, and (f) attention-related sensations from imagined stimulation of two different locations on the fingers. Image sources are (a) from [69] with permission from Wolters Kluwer, (b + f) modified after [37] under CC BY 4.0, (c) modified from [71] under CC BY 4.0, (d) modified after [25] under CC BY 3.0, and (e) modified from [70] by permission of SAGE Publications, Ltd.

A validated and straightforward method for studying the topography of therapeutic sensations is drawings made on a body outline, a modification of the well-known pain drawing method [67, 68]. The systematic literature search for graphical representations of therapeutic sensations (see Supplementary Methods) yielded six articles reporting sensations related to acupuncture [69–72], tactile stimulation [71], low-level laser stimulation [25], and several forms of placebo stimulation [37].  Figure 3 shows a collection of bodily maps of therapeutic sensations reported by the individual studies. It is evident from this comparison that maps from different interventions are similar in that stimulation frequently leads to radiation or spreading of sensations away from the stimulated location. The exact form of radiation and spreading, however, differs between interventions.

### 3.1. Linear, Areal, and Mixed Sensations

**Figure 4:**
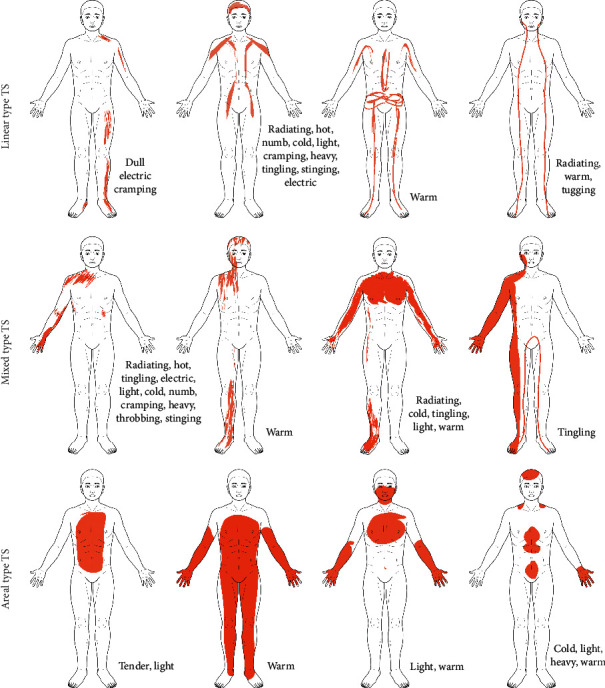
Spatial characteristics of therapeutic sensations patterns as evidenced by digital drawings made by patients following a session of body-oriented psychotherapy combined with acupuncture and related techniques. Note the continuum from linear to areal patterns and the frequent occurrence of paradoxical sensations, like “cold” and “warm” or “light” and “heavy” (modified from [22]).

To better understand the different types of therapeutic sensations, it is helpful to look at individual subjects and the topography of their sensations.  Figure 4 shows a selection of electronic sensation drawings obtained from patients after a session of body-oriented psychotherapy with stimulation by acupuncture, cupping, or moxibustion [22]. Drawings are from a larger sample of 108 patients and have been selected to illustrate the continuum of therapeutic sensations from a *linear type* with a width of a few centimetres to a widespread *areal type* that can cover large parts of the body. The differentiation between *linear-* and *areal-type* therapeutic sensations may be crucial to understand the connection between such sensations and concepts of vital energy, which is discussed in more detail below.

Linear-type therapeutic sensations have received much attention in the field of acupuncture research, where they are called propagated sensations along channels (PSC) and where they have been studied since the early 1950s [73]. Abstracts on PSC submitted to the National Symposia on Acupuncture, Moxibustion, and Acupuncture Anaesthesia held in Beijing in 1979 and 1984 showed for the first time a vast body of research by Chinese scientists (e.g., [74, 75]). Unfortunately, only a fraction of these studies has ever been published in the form of English language scientific papers [69, 72, 76–80] with the rest being inaccessible to Western scientists (for a review, see [81]).

Furthermore, the methodology of many of the Chinese PSC studies (as judged from the available information) leaves much to be desired. What can be said with some certainty from these sources, however, is that subject reports indicate that PSC appear in the form of linear-type therapeutic sensations that propagate with a velocity of 20 cm/s or less and have a width of 1 to 10 cm. Propagation can be stopped by applying pressure or cold [77]. PSC can be elicited in 20 to 89% of subjects using acupuncture and related techniques, while the incidence of marked (i.e., widespread) PSC exceeding three joints is less than 1% [59, 76].

Furthermore, there is a latent form of PSC elicited by electrical stimulation of acupuncture points that follows the same patterns as prominent PSC but only emerges when the skin is additionally stimulated by slight tapping with a hammer [72, 80]. Finally, Xue has shown that PSC can be felt by acquired amputees in the region of the phantom limb (Figure 3(a)) [69], a finding later replicated by Katz and colleagues [26, 82], who reported the induction of phantom limb sensations using TENS of the outer ear. One patient described these sensations as a warm sensation that traveled down his phantom arm and into the hand which then began to swell, while another one described them as tingling in both phantom soles and heels [82].

Despite the methodological shortcomings of early PSC studies, some of their findings have been confirmed by recent studies. These include their linear patterns [25], their propagation to areas remote from the stimulated site [25, 71, 83, 84], and the activation of somatosensory cortical areas by PSC [83]. On the other hand, results on latent PSC and blocking of propagation still await replication.

## 4. The Meaning Dimension of Therapeutic Sensations

The following section examines the meaning dimension of therapeutic sensations, that is, the significance and perceived importance that patients and practitioners attach to them. A systematic literature search (see Supplementary Methods) identified seven studies reporting the results of qualitative unstructured, semistructured, or structured interviews of patients and healthy subjects on their experiences with therapeutic sensations. The studied interventions included acupuncture [3, 59, 85], sham acupuncture [1], massage [86], Reiki [29], and new-age energy healing [87].

**Figure 5:**
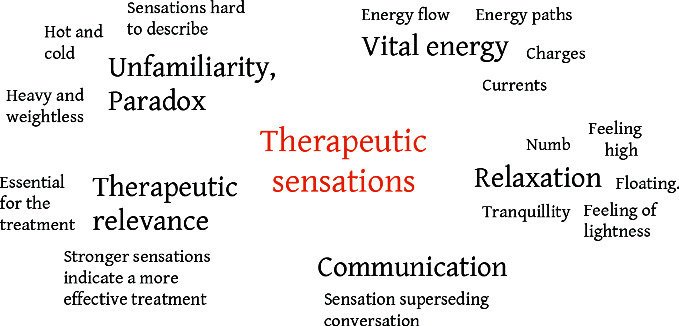
The meaning dimension of therapeutic sensations. Results from qualitative studies based on patient interviews after acupuncture, sham acupuncture, massage, Reiki, and new-age energy healing.

The list of meanings that patients commonly attached to therapeutic sensations is surprisingly long (Figure 5). Some patients were struck by the unfamiliarity of the sensations, making their experience hard to describe accurately [1, 85]. This included paradoxical sensations, like feeling heavy and weightless or hot and cold at the same time, whose conceptualization into the underlying theme of paradox may provide a new perspective [29]. Another common theme was the experience of relaxation, which was equated with “numb” and “floating” sensations, feeling “high,” sleepiness, and tranquillity [85] as well as “floating freely,” “freely in the air,” and a “feeling of lightness” [86]. Kerr and colleagues [1] report a patient, who saw therapeutic sensations as a unique form of communication with his acupuncturist that superseded their conversation and helped her place the needles. The perceived importance of therapeutic sensations for a successful treatment is emphasized by Mao and colleagues [59], where 82 percent of respondents endorsed the statement that acupuncture-related therapeutic sensations are essential for their treatment, and 68 percent believed stronger sensations meant a more effective treatment. The most common theme mentioned in three of the seven studies, however, was the association of therapeutic sensations with concepts of vital energy. Thus, several authors give accounts of patients that equate therapeutic sensations with energy flows [3], energy paths [87], or charges and currents [29].

### 4.1. Interpretations of Therapeutic Sensations in a Body-Oriented Psychotherapy

Many of the topics identified in the systematic literature review have also been covered in a quantitative study that the author conducted in 108 patients receiving body-oriented psychotherapy combined with acupuncture for various conditions (e.g., chronic pain, endometriosis, fatigue, subfertility, epilepsy, and anorexia) [22]. Patients were asked to draw their therapeutic sensations right after the treatment session and to answer the question, “What meaning do these sensations have for you?” (see Table 1).

The results indicate that less than ten percent of the patients saw their therapeutic sensations as normal sensations with no particular meaning attached to them or stated that they did not know what the sensations meant. In stark contrast, the majority of patients in this study associated therapeutic sensations with emotional processes and saw them as an essential part of the therapy and as a sign of the upcoming healing. As in the preceding section, interpretations as vital energy were a common theme, as almost 50 percent of the patients stated that the sensations they experienced were an expression of their vital energy. One-quarter of them even saw therapeutic sensations as a sign that the therapist was guiding her vital energy into them. Interestingly, this did not correspond to the narrative of the treatment.

## 5. Therapeutic Sensations as a Means to Investigate Vitalistic Concepts in Traditional Medicine

In the previous section, we have seen that taking therapeutic sensations as a sign for vital energy processes is one of the most common meanings that patients attach to them. Similar observations have been made by many other scholars before. For instance, Wilhelm Reich states that “subjective vegetative sensations are at the basis of every kind of mysticism, be it Yoga, or the Fascist ‘surging of the blood,' or the reaction of a spiritist medium, or the ecstasies of a dervish” [88]. On a more general note, Hinton and colleagues state that the activation and modulation of sensations have profound effects in relation to meaning and are key dimensions of healing [89].

A particularly intimate connection between therapeutic sensations and vital energy concepts can be found in traditional East-Asian medicine, where most practitioners of acupuncture and moxibustion understand tingling, soreness, distension, and so on as the physiologic equivalent of *deqi* (得氣, “attainment of qi”) and *qizhi* (氣至, “arrival of qi”). Both expressions date back to the earliest classics, namely, the Huang Di Nei Jing [90, 91] and the Nan Jing [92]. They describe the moment when *qi*, the vital energy, arrives at the point where heat was applied or a needle inserted and are considered essential for therapeutic success.

To explore the relationship between therapeutic sensations and vital energy further, we may ask, which specific features of the sensations lead patients and practitioners to believe that what they are experiencing is different from an everyday sensation? Are there parallels between the properties of therapeutic sensations and the typical properties of vital energy postulated by traditional medicine? In the following, let us briefly review some of these properties.

### 5.1. Pneuma, Prana, Qi, and the Subtle Body

The *pneuma* of ancient Greek Medicine, the *qi* of traditional East-Asian Medicine, and the *prana* of South-Asian Ayurveda are three of the most famous examples of vital energy concepts in traditional medicine. Although their origins lie in geographically distinct parts of the world, there are astonishing similarities between the three. For instance, qi, prana, and pneuma are all of an air-like nature, as reflected by their most common translations. While *pneuma* (*πνε*ῦ*μα*) is the ancient Greek word for “breathed air”, “breath”, or “breath of life” [93], the Sanskrit term *prana* (प्राण) means “breath,” “breath of life,” or even “life” itself [94]. The Chinese *qi* (氣) translates as “steam,” “vapor,” or “air,” its character consisting of the radical “air/gas” (气) and the phonetic component “rice” (米) [95], which shows the original meaning of “steam rising from cooked rice.”

Another similarity is that *pneuma*, *prana*, and *qi* are not static, but in constant motion or flow. Corresponding statements can be found in the Corpus Hippocraticum for Greek medicine [96], the Sushruta Samhita for Ayurveda [97], and the Huang Di Nei Jing for traditional East-Asian Medicine [90, 91]. The structures emerging from such motion can be subsumed under the term *subtle body,* a visualized internal structure to the human body that describes a quasi-material level of human functioning [98, 99]. The three systems discussed above postulate that vital energy moves through some form of vessels or channels, that are called *mai* (脈) or jingluo (经络) in traditional East-Asian Medicine, *nadi* (नाडि) in Ayurveda, and *phlebes* (*φλεβες*) in Greek medicine. There is considerable variability regarding the number and course of the channels between the different systems. Nevertheless, several scholars have pointed out similarities for example in the course of the Greek and the Chinese channels [100, 101].

Another important structure of the subtle body in traditional medicine are the so-called *cakras* (चक्र). These are focal points located in the body's midline embedded in the physical body. Their exact number varies between systems [102]. Originating from early traditions of Hinduism, *cakras* have analogues in many other contemplative practices and internal martial arts. For example, East-Asian systems call them *dan tien* (丹田) and they play a vital role in the therapeutic exercises of Qigong and Taijiquan.

A final remark about life energy is that its movement is often associated with the mind. This is least obvious in ancient Greek medicine, where Aristotle, who made important contributions to Greek medical theory, theorized that the *psyche* (*ψυχ*ή), that is, the soul/mind, is what moves the *pneuma.* In the Asian traditions, this association is expressed more clearly in the belief that practitioners of mind-body exercises can learn to direct their vital energy to various places inside their body. Classical texts of Chinese internal martial arts, such as Taijiquan, call this *yi dao qi dao* (意导气导), which translates as “where the mind/intention leads, the *qi* follows” [103, 104]. Similarly, mind and *prana* are inextricably linked in the traditions of yoga and tantra. For instance, the 10th to 11th century Vimānārcanākalpa contains the following description: “After raising the breath through these locations by means of the mind, one should draw it upwards or downwards from [each] location in sequence and hold the breath [there]” [102].

### 5.2. Parallels between Vital Energy and Therapeutic Sensations

Let us now look at the parallels between therapeutic sensations and the vital energy concepts reviewed above (Table 2**)**. The idea of directing vital energy through the body has an obvious parallel in attention-related sensations. In the former, patients or practitioners use their mind to move vital energy to different places inside their bodies [102–104]. In the latter, people report sensations of warmth and tingling, when focusing their attention on certain parts of their bodies [37, 40–42]. Further support for this hypothesis comes from the quote of a meditator [6], who quite naturally equates the “flow of energy” she is directing through her body with the sensation of “a subtle tingling.” Thus, a vitalist and a rationalist may be describing the same experience, when the former speaks of directing vital energy through the body, while the latter reports a tingling sensation when focusing on a particular body region.

Tingling is the most common descriptor for both therapeutic and attention-related sensations and seems to evoke energy-related associations in many people. One reason may be that tingling is commonly elicited by an alternating electrical current of high frequency or by static electricity. Since the latter has been described as early as 600 BCE, we can assume that the connection between this energetic phenomenon and the sensation it evokes has accompanied humans since classical antiquity, which gives a hint, why we equate the two so easily.

Continuing with the somatosensory qualities of therapeutic sensations, the second most common descriptor, warmth, is a general sign of energy and, in the form of body warmth, a core feature of all life. Huang and colleagues even hypothesize that warm sensations experienced during moxibustion may have played a central role in the development of the East-Asian channel concept. This is suggested by the similarity of the glyphs for *wen* 温, meaning “warm” or “moderate temperature,” and the earliest glyph for *mai*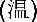, meaning “vessel” [105].

Another group of frequent descriptors of therapeutic sensations are “fullness,” “distention,” and “pressure.” The accumulation or build-up of an unnamed something implicated by these words is paralleled by East-Asian medicine and other traditional systems attributing to vital energy the ability to fill previously empty spaces. Fullness, distention, and pressure are thus plausible ways of perceiving and describing such accumulation of energy.

The focus on individual descriptors should not disguise the fact that patients frequently need five or even ten descriptors to describe their therapeutic sensations adequately. Thus, we are dealing with complex sensations that are hard to describe and often reflect the entire bandwidth of possible somatic sensations [37]. Furthermore, paradoxical sensations, like “warm and cold” or “heavy and light,” are very common and, as discussed above, often unfamiliar to the person experiencing them. A quote from the introduction reflecting this comes from a patient describing the sensations during his acupuncture treatments as “*quite a bizarre sensation that I've never had before*” [3]. Their complexity and unfamiliarity set therapeutic sensations apart from any ordinary sensation. As such, under the right circumstances, they may reinforce the slightest pre-existing impression that an exceptional event, such as energy exchange, is taking place.

Another parallel between therapeutic sensations and vital energy concepts is nonstationarity, that is, tendency to move. This is paralleled by “flowing” or “streaming” being common descriptions of therapeutic sensations in qualitative studies. As discussed above, *pneuma*, *prana*, and *qi* are not static, but in constant motion or flow. Similarly, “flowing” or “streaming” are common ways, how people describe therapeutic sensations in qualitative studies. Furthermore, other common descriptions, such as “spreading” or “radiating,” seem to depict movement inside the body, just like the constant flow attributed to vital energies, moreover, just like warmth movement is a core feature of life.

**Figure 6:**
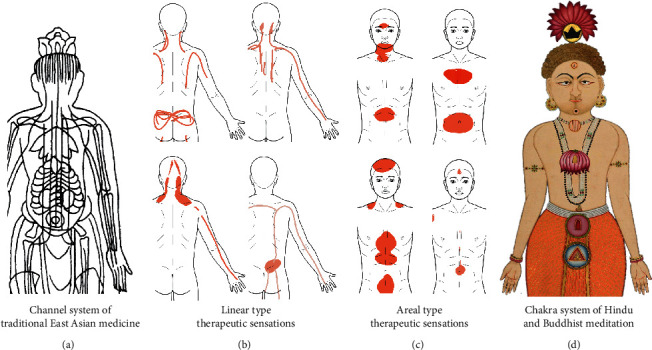
Comparison of common therapeutic sensations patterns with different concepts of vital energy in traditional medicine and spirituality from the east. (a) Traditional channels (jingluo) of traditional East-Asian medicine from the Illustrated Canon of Acu-moxa by Wang Weiyi, carved on a stone stele in Song dynasty, (b) electronic drawings by four different patients with linear-type therapeutic sensations in the arm/shoulder region, (c) electronic drawings by four different patients with areal-type therapeutic sensations in the body midline, and (d) chakras of Hindu and Buddhist meditation as illustrated in the Siddha Siddhanta Paddhati by Bulaki, India, Rajasthan, Jodhpur, 1824. The different forms of therapeutic sensations may have inspired both the jingluo and chakra concepts. Image sources are (a) modified after [106], (b, c) modified from [22], and (d) courtesy of Mehrangarh Museum Trust.

Further exciting parallels that we are just beginning to understand exist between the topography of therapeutic sensations and some aspects of the subtle body. A central observation is that linear-type therapeutic sensations bear many similarities with the concept of channels (Figure 6). Several experiments have shown that the pathways of linear-type therapeutic sensations evoked by acupuncture and related techniques are very similar to those of the traditional channels, which has led to the term *propagated sensations along channels* [72, 76, 80, 81]. In particular, Beissner and Marzolff have shown that laser stimulation of acupuncture points under sensory deprivation can evoke linear sensations that follow the course of traditional East-Asian channels with an average distance of around one centimetre [25]. It is thus straight forward to assume a direct relationship between the various channels concepts found in traditional medicine and linear-type therapeutic sensations, an idea that has been brought forth by several other scholars before (cf. [107, 108]). To bring the idea to full fruition, however, it is necessary to look past the modern versions of the channels with their zigzag patterns and instead use the older versions, such as those displayed in the Illustrated Canon of Acu-moxa by Wang Weiyi from the year 1029 [109] (Figure 6).

Just like the linear-type therapeutic sensations, those of the areal type also have their counterpart in the subtle body, namely, the *cakras*. This connection has been even less scientifically researched. Still, as Figure 6 shows, the similarity of *cakras* with areal-type therapeutic sensations occurring in the midline of the body is hard to ignore.

Finally, there are also instances, in which the practitioner experiences therapeutic sensations. They usually follow the narrative of “vital energy exchange” in therapeutic or spiritual encounters. Here, vital energy is believed to leave the body of one person (healer) and be transmitted directly to another one (client) for instance, by laying on of hands. However, the experience of vital energy is still limited to the body as evidenced by two quotes from the beginning of the article. Here, one client reports that the healer “was chasing wind round my body” [4], while a nursing student treated by the same method “*felt the heat coming out of the therapist's hands*” [2]. Furthermore, the healer notices the energy exchange by very similar sensations in his or her body, as noted by Csordas, who reports that Catholic Charismatic healers feel tingling, heat, or outflow of “power” similar to an electrical current, often in the hands, but at times in other parts of the body [31]. Thus, the same mechanism that generates therapeutic sensations in therapeutic encounters may be at work in both, the healer's and the client's body.

## 6. Conclusions

Therapeutic sensations are ubiquitous in mind-body interventions and exercises. The similarity of their topography and somatosensory qualities suggests that they are a universal phenomenon that can be studied across the boundaries of the individual interventions. Furthermore, their close connection with vital energy concepts makes them an ideal starting point to study such concepts scientifically.

The following list contains some of the most urgent unresolved research questions, the answers to which have the potential to significantly improve our understanding of therapeutic sensations. Of course, it is not exhaustive.

- Can we confirm the hypothesis that the somatosensory qualities of therapeutic sensations are similar across interventions and exercises? How can we construct a suitable questionnaire for such a task and for the study of therapeutic sensations in general?
- Are there other important sensory aspects of therapeutic sensations that we might have missed due to the lack of descriptor-based questionnaires for (innocuous) tactile, haptic, proprioceptive, and visceral sensations?
- Are there differences (e.g., regarding quality and topography) between therapeutic sensations elicited by invasive and noninvasive interventions? Is there a special role for painful sensations?
- Do linear-type and areal-type sensations differ in their somatosensory qualities?
- What are the temporal dynamics of therapeutic sensations? At what speed do linear-type therapeutic sensations propagate? Can the propagation be blocked (e.g., by cold or pressure) as suggested by the PSC literature?
- Is there a latent form of therapeutic sensations as suggested by the PSC literature?
- Is it possible to construct a map of the subtle body (i.e., channels and cakras) based on sensation drawings? Would practitioners from different systems agree on such a map?
- What is the affective dimension of therapeutic sensations? Are the sensations emotionally coloured?
- To what extent do sensory qualities, topography, and contextual factors influence the meaning that people attach to therapeutic sensations?
- What are possible mechanisms by which therapeutic sensations exert their clinical effect?

To conclude, therapeutic sensations are a fascinating object of study at the boundary between medicine, neuroscience, anthropology, and ethnology. Unlike previous attempts that have tried to measure a physicalized version of vital energy, therapeutic sensations offer a means to study this phenomenon by focusing on the human being and may thus help to find a common language between scientists, patients, and practitioners.

## Figures and Tables

**Table 1:** Significance of therapeutic sensations for patients experiencing them (from [22]).

Answers to the question: “What meaning do these sensations have for you?”	*n* (%)
“They are a bodily expression of emotional processes.”	93 (62%)
“They are an important part of the therapy.”	78 (52%)
“They are an expression of my life energy.”	69 (46%)
“They mean that I am being healed.”	57 (38%)
“The therapist is guiding her life energy into me.”	33 (22%)
“They are only sensations.”	13 (9%)
“I do not know, what they mean.”	10 (7%)

**Table 2:** Conceptual parallels between therapeutic sensations and vital energy.

Characteristic of therapeutic sensations	Reference to vital energy
*General properties*	
Attention-related sensations	Directing vital energy through the body
*Somatosensory quality*	
Tingling, electric	Electricity as a form of energy
Warmth	Body warmth as a core feature of life
Fullness or distention	Energy accumulating in a body region
Complex sensations	Unfamiliarity/exceptional experience
*Nonstationarity, nonlocality*	
Flowing, streaming	Energy flow
Radiating, spreading	Movement as a core feature of life; energy flow
*Topography*	
Linear sensations	Energy moving through channels
Widespread sensations in body midline	Energy accumulating in chakras

## Data Availability

All data associated with this article are available from the author upon reasonable request.

## Supplementary Materials

Supplementary Materials Supplementary Methods: methodological details on the three systematic literature searches (Somatosensory qualities, topography, and meaning dimension of therapeutic sensations). Supplementary Table 1: results of the literature search for descriptors of therapeutic sensations in different interventions. Descriptor frequencies were first summed up for each intervention and then for all interventions. Please note that a frequency of zero (“0”) in this table means that study participants were asked for the particular sensations but did not experience it.
