# Supplementary material for: Therapeutic Sensations: A New Unifying Concept
Source: Evid Based Complement Alternat Med. 2020 Aug 6;2020:7630190. doi: 10.1155/2020/7630190 (PMC7428881; doi:10.1155/2020/7630190)
Supplement: Supplementary Materials — Supplementary Methods: methodological details on the three systematic literature searches (Somatosensory qualities, topography, and meaning dimension of therapeutic sensations). Supplementary Table 1: results of the literature search for descriptors of therapeutic sensations in different interventions. Descriptor frequencies were first summed up for each intervention and then for all interventions. Please note that a frequency of zero (“0”) in this table means that study participants were asked for the particular sensations but did not experience it. [file 7630190.f1.zip › 7630190.f1/Supplementary Methods.docx]

# Supplementary Methods

## Systematic literature searches

This review article combines systematic and narrative elements. For each of the three main topics (somatosensory qualities, topography, and meaning dimension) a systematic literature search was conducted.

Literature searches were conducted in Google Scholar (http://scholar.google.com), MEDLINE (https://www.ncbi.nlm.nih.gov/pubmed), and PsycNet (http:psycnet.apa.org) including all literature published until April 24th, 2020 with no start date specified.

### Somatosensory qualities of therapeutic sensations

Relevant articles were identified by searching for various therapeutic interventions in the title combined with the search pattern (”sensation” OR ”sensations”) AND (”items” OR ”descriptors”) in the main text. For example, the exact search pattern for acupuncture was intitle:”acupuncture” AND (”sensations”) AND (”items” OR ”descriptors”). Where restriction to the title was not possible, the search pattern was applied to all fields instead.

This approach yielded 423/6/0 articles in Google Scholar/MEDLINE/PsycNet for “acupuncture,” 38/0/0 for “acupressure” OR “shiatsu,” 9/0/0 for “cupping,” 105/0/7 for ”focused attention” OR ”bodily attention” OR ”attention-related”, 114/1/0 for “massage,” 23/1/0 for “moxibustion,” 372/1/3 for “placebo,” 86/0/0 for ”TENS” OR ”transcutaneous electrical nerve stimulation” OR ”electrostimulation,” 92/0/0 for ”therapeutic touch” OR ”touch healing” OR ”healing touch” OR ”Reiki”, 38/0/0 for “qi gong” OR “qigong” OR “chi kung” OR “chi gung,” 34/0/0 for “taiji” OR “tai ji” OR “tai chi” OR “taichi,” 636/0/2 for “yoga,” and 111/1/0 articles for ”ultrasound”.

All abstracts were screened, and articles included if they (1) reported sensations perceived by patients or subjects during therapeutic interventions or exercises, (2) reported frequencies of individual descriptors for these sensations, and (3) presented results in tabular form. The tabular form was required since plots do not allow to extract exact values. Studies were excluded if they reported results from brain imaging experiments (i.e., fMRI, EEG, or MEG), since the specific conditions in such experiments (e.g., scanner noise, electrodes attached to the body, or uncomfortable positions) may considerably influence perception. Furthermore, review articles and articles without peer-review, like books and dissertations, were excluded.

The analysis consisted of extracting descriptors of somatosensory sensations together with their respective frequencies, while excluding evaluative or affective descriptors. If more than one study was available, frequencies from the single studies were added up. For details and the complete list of descriptors, see Supplementary Table 1. Please note that a frequency of zero ("0") in this table means that study participants were asked for the particular sensations but did not experience it.

Results for the individual interventions were converted into word clouds, where font size represents frequency using the generator at http://worditout.com. This type of presentation allows for a direct and intuitive comparison of the different sensations. In a final step, the average descriptor profile of TS was calculated by adding up the frequencies of descriptors from the eight individual interventions. This simple procedure gives higher weight to well-powered studies. For this, it was necessary to harmonize descriptors (e.g., by summarizing “warm” and “warmth” under “warm”).

In total, 18 articles were included in the analysis, many of which appeared in more than one search [11, 20, 23, 25, 35, 37, 42, 50, 57-66].

### The topography of therapeutic sensations

The approach used for the analysis of bodily maps of TS was similar. Here, the literature search identified relevant articles by the search terms ("sensation" OR "sensations") in the title and ("pattern" OR "patterns" OR "map" or "maps") in the main text. These terms were combined with an OR-separated list of all therapeutic interventions used in the first literature search (see above).

Articles were included if they (1) reported sensations perceived by patients or subjects during therapeutic interventions or exercises, and (2) contained a graphical representation of TS on an outline of the human body or parts thereof. Once again, brain imaging studies, reviews, or non-peer-reviewed articles, like books and dissertations, were excluded.

Due to restrictions in the maximum search string length, the search had to be split up in three yielding 277/9/4, 474/10/8, and 224/3/5 articles respectively, which partially overlapped.

Altogether, the final analysis comprised six studies [69-72].

### The meaning dimension of TS

For the meaning dimension of TS, the focus lay on qualitative studies applying patient interviews. Therefore, an OR-separated list of therapeutic interventions was searched for in the title combined with the search pattern (”sensation” OR ”sensations”) AND ”meaning” AND ”interview” in the text body. Articles were included if they (1) reported sensations perceived by patients or subjects during therapeutic interventions or exercises, and (2) if their results were based on patient interviews and contained statements on the meaning of sensations experienced during interventions or exercises.

Once again the search had to be split up (with overlapping results) yielding 184/0/10, 222/1/3, 165/0/2, and 516/0/2 articles.

Here, the literature search identified seven studies fulfilling the inclusion criteria [1, 3, 29, 59, 85-87].
